# Helping to improve demand for mental health services: Reach and adoption of a community mental health education and detection tool by community health workers within routine care in South Africa

**DOI:** 10.1017/gmh.2026.10256

**Published:** 2026-06-24

**Authors:** Merridy Grant, Tasneem Kathree, Londiwe Mthethwa, Gillian Faris, Gcina Radebe, Arvin Bhana, André Janse van Rensburg, Zamasomi Prudence Busisi Luvuno, Nikiwe Hongo, Inge Petersen

**Affiliations:** 1 Curtin EnAble Institute, Curtin University, Australia; 2 Centre for Research in Health systems, School of Medicine, University of KwaZulu-Natal, Durban, South Africa; 3 Gill Faris, Cape Town, South Africa; 4 Health Systems Research Unit, South African Medical Research Council Durban, South Africa; 5 Mental Health Directorate, KwaZulu-Natal Department of Health, South Africa; 6 University College London, UK

**Keywords:** detection, referral, mental health, community health workers, intervention scale-up

## Abstract

The Community Mental Health Education and Detection (CMED) tool was designed and validated for community health workers (CHWs) in South Africa to promote mental health education, detection and linkage to care for adults at risk of mental health conditions. This study evaluated CMED scale-up using implementation research to understand reach and adoption.Routinely collected CHW data from three scale-up community areas were analysed over six months. Using the Reach and Adoption components of the RE-AIM framework, data included the (i) number of CMED administrations; (ii) proportion of identified presumptive cases; and (iii) proportion of referred cases who received care. These data identified high-and low-adopting CHW teams. Observations and repeated group discussions explored factors influencing adoption. CHWs completed 2,135 CMED administrations. Seventeen percent screened positive and were referred for further assessment at PHC facilities; 62% of those referred presented for assessment, diagnosis, and management. Adoption varied across teams. Barriers included poor data systems and inconsistent supply of mental health services. Supportive leadership and supervision were strong facilitators of adoption. Policy uptake signalled maintenance. Findings suggest the CHW-delivered CMED tool is viable and useful for narrowing the treatment gap by strengthening demand for and access to mental health services.

## Graphical abstract

**Figure:**
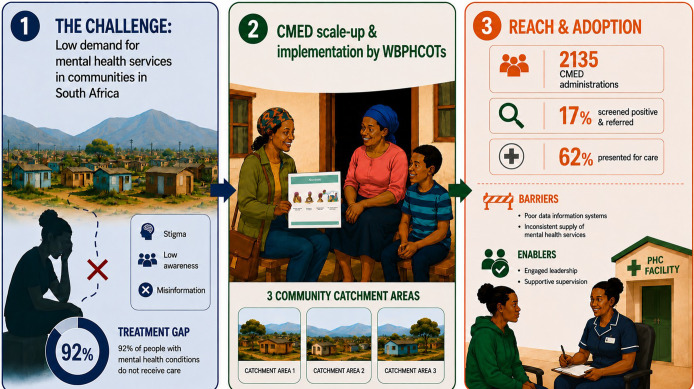

## Impact statement

Early identification and linkage to care at the community level through the community health worker-delivered CMED tool shows potential to help narrow the mental health treatment gap in South Africa and other LMICs.

## Introduction

Globally, a substantial treatment gap persists for common mental disorders (CMDs), with the disparity most acute in low- and middle-income countries [LMICs] (Moitra et al., 2022; Vigo et al., 2022; World Health Organization, 2022). In South Africa, the treatment gap for CMDs is estimated at 92%, with only 7% of the uninsured population receiving any form of care (Docrat et al., 2019). Addressing this gap requires not only increasing the supply of mental health services through a task-sharing approach (Patel et al., 2016) but also strengthening demand for services (Jordans et al., 2020). Many individuals experiencing mental health symptoms do not seek care due to limited awareness of symptoms and available services, as well as pervasive stigma and misinformation surrounding mental illness and its treatment (Subba et al., 2017; Grant et al., 2021). In addition, the symptoms of mental health conditions themselves such as low motivation or social withdrawal may impede help-seeking (Mohr et al., 2006). Structural barriers, including limited access to appropriate, affordable, high-quality and responsive services, further constrain individuals’ ability to seek care (Semrau et al., 2015; Thornicroft et al., 2017; Patel and Farmer, 2020). To date much of the evidence has focused on strengthening supply of services, with very few examples of evidence-based tools in LMICs designed to increase demand for mental health services.

This study was nested within a broader implementation research study that aimed to iteratively evaluate and strengthen a systems-level implementation package. The goal was to optimise the real-world delivery of evidence-based, integrated collaborative care for common mental health conditions within primary health care (PHC) services in South Africa (Petersen et al., 2021; Petersen et al., 2023). Known as the Mental health INTegration (MhINT) package, implementation strategies include, inter alia, co-developed educational materials, training, workflow redesign, ongoing consultation and facilitation, as well as audit and feedback through continuous quality improvement (CQI) (Petersen et al., 2021). Key elements of the initial MhINT implementation package, tested in the Amajuba District in KwaZulu-Natal (KZN), South Africa, included building the capacity of professional nurses to identify chronic care clients with comorbid CMDs at PHC facilities, and strengthening referral pathways depending on symptom severity – either to facility-based lay counsellors for counselling using cognitive-behavioural therapy techniques or doctors for initiation of psychotropic medication or mental health specialists for more complex care (Petersen et al., 2021).

The first-stage evaluation of the MhINT package identified gaps in the identification of CMDs at a community level and in community members’ understanding of symptoms of mental health conditions and how to seek help (Grant et al., 2021; Grant et al., 2023; Petersen et al., 2023). In response, the KwaZulu-Natal Department of Health (KZN DoH) requested the Centre for Research in Health systems team at the University of KwaZulu-Natal (UKZN) to expand the MhINT package to include, inter alia, resources for ward-based PHC outreach teams (WBPHCOTs) to provide education on mental health symptoms, identify and refer community members with possible mental health conditions for further assessment, and provide mental health promotion information during routine household visits (Grant et al., 2021). This led to the development and assessment of the Community Mental Health Education and Detection (CMED) tool (Grant et al., 2021).

Ward-based PHC outreach teams are an essential component of PHC re-engineering in South Africa (Pillay and Baron, 2011; Assegaai and Schneider, 2019). They comprise teams of community health workers (CHWs), supervised by Outreach Team Leaders (OTLs) who are either professional or mid-level enrolled nurses. CHWs, who typically reside in the communities they serve, are required to have a minimum of a Grade 10 education supplemented by basic training (South African National Department of Health, 2017). WBPHCOTs are linked to designated PHC facilities and service municipal wards. Their role is to provide integrated health services at the community level, with a focus on health promotion, prevention, adherence counselling and monitoring, risk screening and linkage to PHC facilities for care through household visits and other community-based platforms (Assegaai and Schneider, 2019). The CMED tool was designed to provide a single culturally and contextually appropriate resource that WBPHCOTs could use to perform these mental health functions, alongside physical health services – covering mental health promotion (increasing mental health awareness, psychoeducation and self-care), mental health risk screening and linkage to care (Grant et al., 2021).

The first-stage evaluation was conducted with one large WBPHCOT in KZN, South Africa. It demonstrated that the CMED tool was valid, culturally and contextually appropriate and feasible for use by WBPHCOTs (Grant et al., 2022; Grant et al., 2023). It also demonstrated that the CMED enabled mental health to be practically integrated at a community level as part of primary mental health care services through a task-sharing approach. This paper reports on a second-stage evaluation of the CMED, assessing its Reach and Adoption in the context of real-world scale-up to WBPHCOTs servicing one district in South Africa.

## Methods

### Approach

An observational research design using a pragmatic application of the RE-AIM implementation outcome framework (Glasgow et al., 2013), previously used in the main study (Petersen et al., 2023), was adopted to assess Reach and Adoption. Assessment of Effectiveness was not possible under real-world conditions. In terms of Implementation, a mentoring and support tool was developed to build confidence and support the delivery of all CMED components. This tool was used qualitatively during mentoring visits rather than as a quantitative measure, limiting the ability to formally assess implementation fidelity. Assessment of Maintenance was inhibited by funding and time constraints requiring a lengthy follow-up period to assess continued use of the tool, although we do report on adoption of the tool into policy. Routinely collected data were used to examine Reach and Adoption components as they pertain to household-level identification, referral and access to mental health and psychosocial services as part of routine care.

### Setting

The second-stage evaluation was conducted in the Amajuba District in KZN, South Africa. The district had a population of approximately half a million people, spanning urban, peri-urban and rural communities serviced by 24 PHC facilities (KwaZulu-Natal Department of Health, 2016). Eligibility criteria required that community catchment areas or wards included in the study be serviced by fully constituted WBPHCOTs. The latter were defined as CHW teams with a designated OTL – either a registered or enrolled nurse. Nine community catchment areas/wards and associated WBPHCOTs met these criteria.

### Description of the scale-up package

#### CMED tool

**Figure 1.**
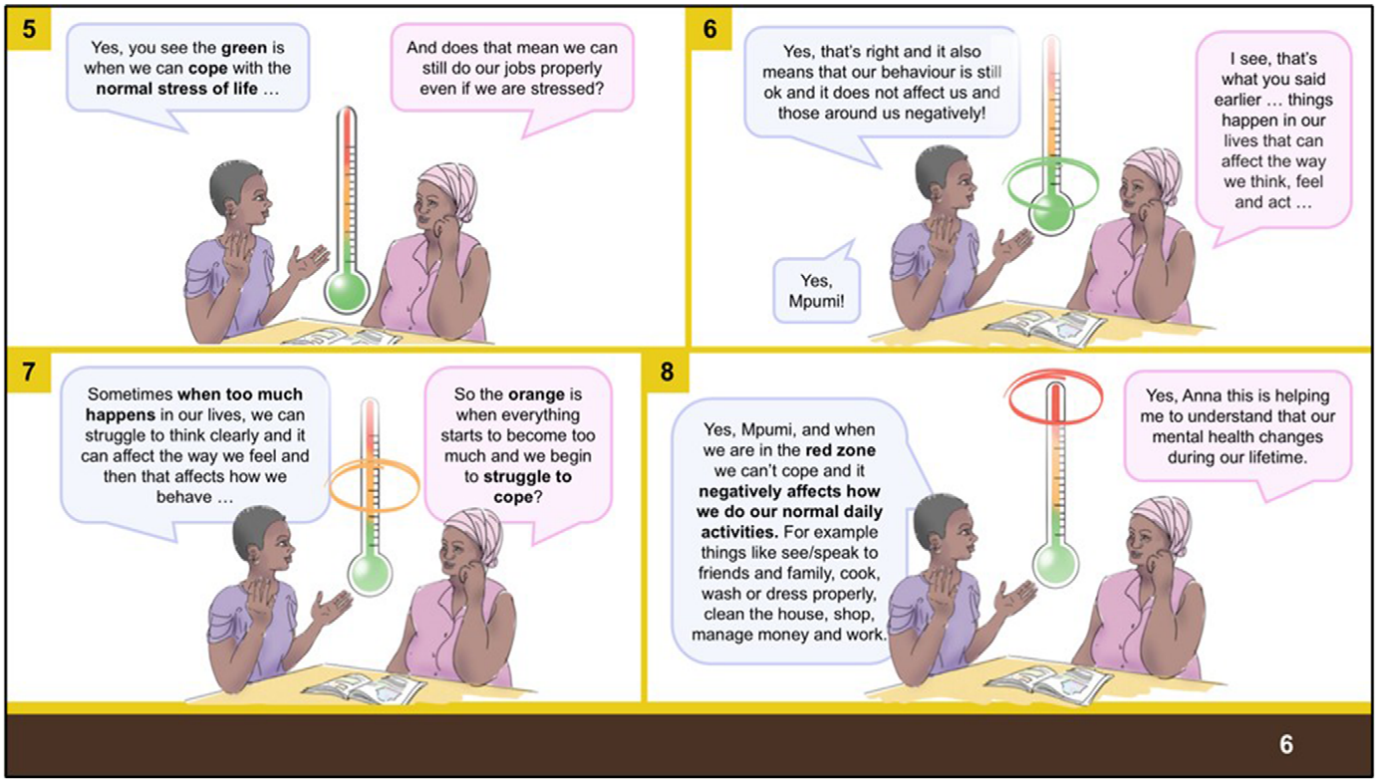
Example of the emotional health thermometer. Figure 1. long description.

The CMED tool was based on a prototype matching approach, in which symptoms were presented through five brief case vignettes (prototype paragraphs), each accompanied by related illustrations, to facilitate the detection of possible depression, anxiety, psychosis and harmful alcohol and drug use, and to support appropriate referral for further assessment. A mental health flowchart with guiding questions prompted CHWs on which vignette to read to the household. The CMED also included mental health education and healthy lifestyle advice. Since the previous iteration of the tool, the educational component of the CMED was expanded to include a vignette using the analogy of an emotional health thermometer to explain mental health (Figure 1). Further detail about the CMED tool is included in the Supplementary Material.

#### Training package design

The training design was embedded in the principles and practices of adult learning and teaching, drawing on socio-cultural theory (Vygotsky, 1978; Engeström, 2014), communities of practice (Lave and Wenger, 1991), experiential learning (Kolb, 2014), reflection IN action (Schön, 2017) and transformative learning (Mezirow, 2018). Through integrating the use of these theoretical approaches, the instructional tools were designed to encourage and promote autonomous practice. The content weaves understanding of mental health conditions with self-reflection and self-care to ensure that those receiving the training are not overwhelmed and can identify and be supported to manage personal mental health challenges that emerge during the training and during the administration of the CMED.

The CMED training used a cascade model to scale up implementation in the designated sites. In this approach, CMED Master Trainers from the UKZN team trained OTLs to train and provide mentorship support to their CHW teams. The instructional tools thus considered that those facilitating the programme were not mental health specialists, educators or trainers, which meant that the instructions needed to be self-explanatory, incorporating theories and concepts into the text of the instructional design of the resources. The training resources for each level of the cascade form a coherent suite of materials designed to capacitate the end user to provide comprehensive and standardised mental healthcare within the health system. Materials used at the CHW and household level were translated into *isiZulu* to standardise and localise the language.

### OTL training – Part one

OTLs from the nine WBPHCOTs servicing the nine wards attended two 1-day trainings on the CMED. Delivered by CMED Master Trainers, these sessions prepared OTLs to train their CHWs to use the tool during routine household visits.

The first training, conducted at a central venue, introduced the 27 OTLs to the CMED tool that CHWs would use in the household to provide mental health education, risk screening, healthy lifestyle advice and where needed, facilitate referral to the next level of care. The training was also attended by representatives from the National Department of Health (NDoH), the KZN DoH and the Amajuba District Health Office, who observed the sessions. This ensured alignment of the training and the CMED tool with national and provincial WBPHCOT policy and supported understanding of the operational requirements for scaling up implementation across KZN and other provinces.

The second OTL (*n* = 27) training was decentralised and focused on preparing OTLs to train their CHW teams in the use of the tool at a household level. OTLs were provided with a facilitator’s guide to standardised training delivery, which also included components on self-care and self-reflection, and supported their role in mentoring and supervising CHWs.

### CHW training

Following the OTL training, OTLs coordinated and delivered a 2-day training of their CHWs. The training covered basic mental health concepts, use of the CMED tool and the importance of CHW self-care (Grant et al., 2023). CHWs were equipped to provide psychoeducation and health promotion, conduct risk screening, and facilitate referral to PHC facilities for further assessment and diagnosis by trained nurses. They were also encouraged to engage in self-reflection and identify ways to improve their practice with support from their OTLs. Given their generalist role, CHWs were prompted to consider how mental health could be integrated into their routine household visits.

The training was supported and, in some facilities, co-facilitated by the CMED Master Trainers. Co-facilitation occurred when OTLs requested assistance due to a lack of confidence in independently delivering the training, helping to optimise fidelity. A total of 245 CHWs were trained by OTLs between November 2021 and January 2022.

### Mentoring and support

All trained OTLs were given the opportunity to organise three or more household visits with their CHWs immediately after completing training. These visits served as mentoring and support platforms, enabling the CMED Master Trainer to assess whether OTLs and CHWs were able to apply their training with fidelity. They also provided an opportunity for the Master Trainer to observe and follow up on clients identified and referred to clinics for mental healthcare. In addition, the Master Trainer conducted mentoring visits with OTLs and CHWs, during which a mentorship and support tool was used to scaffold learning and strengthen CHWs’ confidence in delivering the CMED.

### Work-integrated learning – Part two

Work-Integrated Learning (WIL) refers to weekly in-service sessions that provide an opportunity to reinforce core skills learned during classroom training, identify knowledge and skill gaps, and activate plans to address areas requiring further learning (Berndtsson et al., 2020).

A WIL programme comprising seven sessions was developed to help OTLs in providing debriefing and emotional support to their CHW teams, strengthen CHWs understanding of the main mental health conditions included in the CMED and introduce new content. New content included care for older adults with memory loss, as well as support for adherence to treatment of common NCDs such as HIV, TB and diabetes.

**Figure 2.**
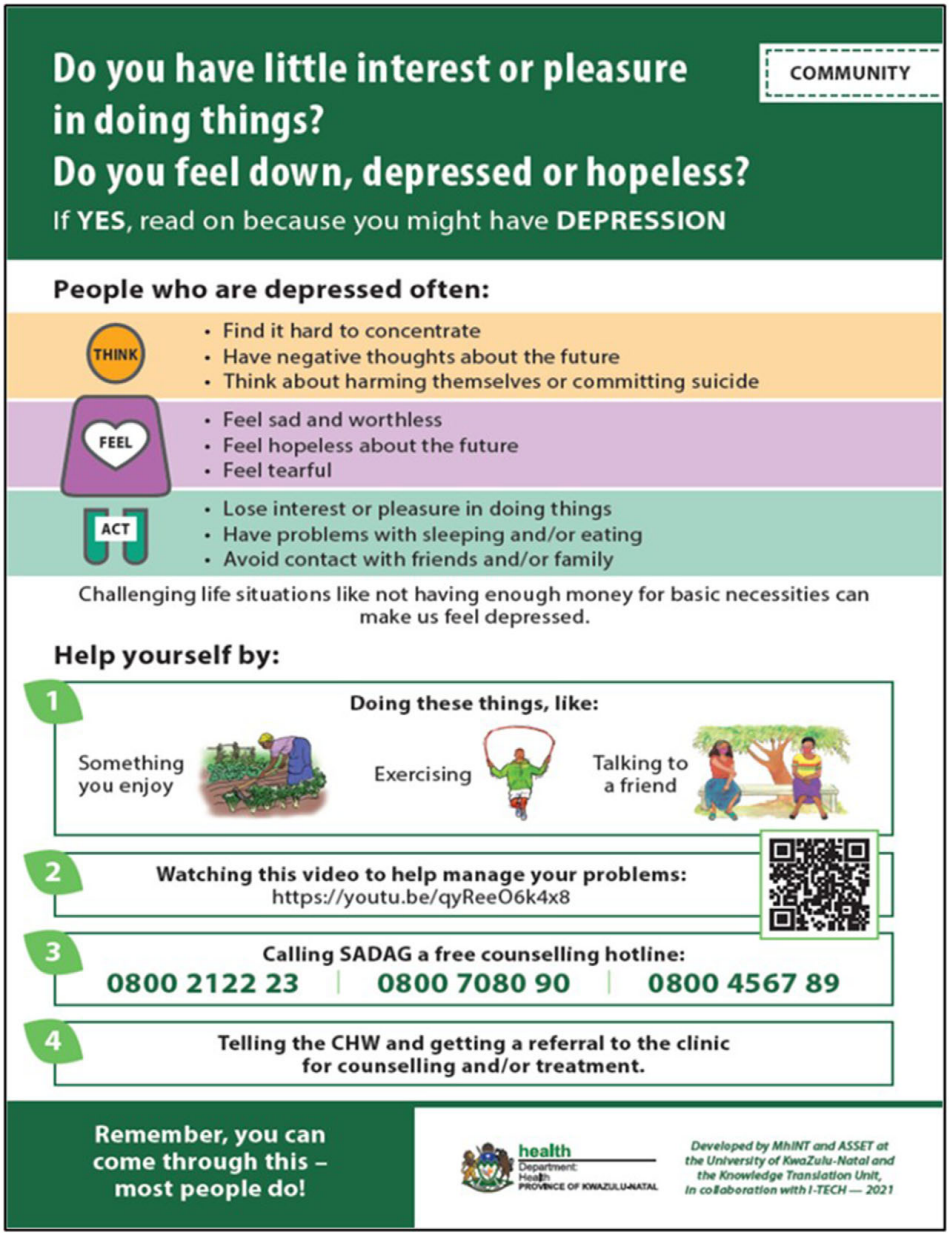
Example of the stepped-up health promotion information provided. Figure 2. long description.

The programme also incorporated a stepped-up approach to health promotion for conditions covered by the CMED. In addition to healthy lifestyle information, this approach included links to self-help strategies, problem solving skills, information on counselling hotlines and guidance on accessing care within the health care system (see Figure 2 for an example). The WIL programme was introduced to the OTLs through a 1-day training programme, after which OTLs conducted WIL sessions with their CHW teams.

A description of the nine trained WBPHCOTs is provided in Table 1.

**Table 1.** Number of WBPHCOTs trained in CMED per facility Table 1. long description.

WBPHCOT	Number of OTLs	Number of CHWs
WBPHCOT One*	1	24
WBPHCOT Two*	2	36
WBPHCOT Three*	1	9
WBPHCOT Four	4	9
WBPHCOT Five	7	100
WBPHCOT Six	4	6
WBPHCOT Seven	3	15
WBPHCOT Eight	3	18
WBPHCOT Nine	2	28
	**27**	**245**

* Facilities included to evaluate reach.

### Data collection

Reach was assessed along the care cascade at both the community level (CMED administration and referrals) and the facility level (referrals and presentation for care). Adoption was assessed by calculating the average number of CHWs in each WBPHCOT who were trained to use the CMED tool.

To evaluate the Reach of the CMED intervention by CHWs, we conducted a review of routine data collected by CHWs along the PHC mental health care cascade between February and July 2022. This time frame was selected as all OTLs and CHWs completed their training in January 2022, and a 6-month implementation period was considered sufficient for embedding the intervention.

Community health workers manually documented household visit activities in notebooks as part of their routine recordkeeping. In addition, they were asked to record: (i) the number of household visits in which the CMED tool was administered; (ii) the number of individuals who screened positive for potential mental health concerns and were subsequently referred for further care, including the type of referral made; and (iii) whether referred individuals presented at a PHC facility for follow-up. This follow-up information was obtained through CHW household visits to confirm whether referrals were acted upon and to understand the type of referral received.

Variation in the Adoption of the CMED intervention was assessed through detailed observation of household visits and recordings of the OTL-delivered CMED trainings and WIL sessions documented by the CMED Master Trainer in an electronic fieldwork diary. Qualitative data were also collected from notes of weekly CHW reflective discussions on the use of the CMED held with the OTLs and the CMED Master Trainer. These data were supplemented with facility profiles to provide the context of the clinics to which the WBPHCOTs were linked.

### Analysis

Community health workers and OTLs submitted CMED-related data to the CMED Master Trainer for entry into a database. Data were cleaned on an ongoing basis with monthly summary reports. Missing data were verified with CHWs and resolved where possible. Referral notes were mostly contained in unstructured data reports. These notes were reviewed for clarity and accuracy, coded into categories, quantified, and analysed using descriptive statistics.

Complete data along the entire care cascade were only available for three community catchment areas serviced by three WBPHCOTs comprising 69 CHWs in total (Table 2). CHWs are responsible for a predetermined number of households allocated to them as part of their duties, with a total of 6,986 households allocated to the 69 CHWs across these three community catchment areas. The qualitative data were analysed using the Framework Method (Gale et al., 2013), which enabled the identification of both *a priori* and inductive themes. Electronic fieldwork diary entries and group reflections were uploaded into NVivo (version 14) and analysed through the five stages of the Framework Method (Gale et al., 2013). This included familiarisation, development and application of a thematic framework (indexing), construction of a summarised matrix for each theme (charting), and overall interpretation of findings. The research team met weekly to review the thematic framework and resolve any discrepancies.

**Table 2.** Reach across the care cascade for three community catchment areas Table 2. long description.

	Feb 22	Mar 22	Apr 22	May 22	Jun 22	July 22	Totals
**Referrals from community to PHC facility level**							
Total household visits	1911	2,383	2,120	2,702	2,621	2,525	14,262
CMED visits	301 (16%)	468 (20%)	378 (18%)	427 (16%)	238 (9%)	323 (13%)	2,135 (15%)
Clients referred using CMED	26 (9%)	59 (13%)	38 (10%)	45 (10%)	33 (14%)	159 (49%)	360 (17%)
Clients presenting at clinics after referral	19 (73%)	39 (66%)	32 (84%)	35 (78%)	25 (76%)	72 (45%)	222 (62%)
**Onward referrals from the PHC nurse**							
Clients referred to the PHC counsellor	13 (68%)	24 (62%)	23 (72%)	22 (63%)	10 (40%)	15 (21%)	107 (48%)
Clients referred to social services	2 (11%)	11 (28%)	4 (13%)	6 (17%)	7 (28%)	33 (46%)	63 (28%)
Clients referred to the hospital	0 (0%)	0 (0%)	1 (3%)	2 (6%)	4 (16%)	5 (7%)	12 (5%)
Psychologist	0 (0%)	1 (3%)	0 (0%)	3 (9%)	2 (8%)	0 (0%)	6 (3%)
Rehab	0 (0%)	1 (3%)	0 (0%)	0 (0%)	1 (4%)	1 (1%)	3 (1%)
Doctor	0 (0%)	0 (0%)	0 (0%)	0 (0%)	0 (0%)	1 (1%)	1 (0.45%)
Other	0 (0%)	0 (0%)	0 (0%)	0 (0%)	0 (0%)	1 (1%)	1 (0.45%)

## Results

### Reach

**Figure 3.**
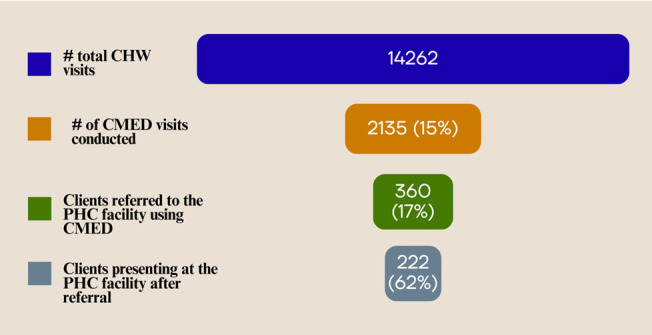
Reach across the care cascade from community to PHC facility in three community catchment areas. Figure 3. long description.

The WBPHCOTs included in the study recorded a total of 14,262 routine home visits between February and July 2022. During these visits, CHWs recorded administering the CMED tool in 2,135 households, resulting in 360 household members being detected as having a possible mental health condition and referred to PHC facilities. Routine CHW follow-up data indicated that 222 of these referred clients acted on the referral and presented at PHC facilities for further assessment and care (see Table 2 and Figure 3).

On presentation at the PHC facilities, all 222 clients were consulted by a Registered Nurse as part of the routine care cascade. Eight clients did not receive further intervention beyond the nurse consultation; however, half (*n* = 4) were reported to have received counselling during the consultation. A further 33 clients (15%) were reported to have received prescribed medication. Specific details regarding the medications were unavailable, and it is presumed that these prescriptions did not involve the initiation of psychotropic medication, as nurses in South Africa are not authorised to prescribe such medication. Additionally, 107 clients (48%) were referred to the PHC counsellor. Records indicated that one client was not assisted due to the counsellor being unavailable, while another was scheduled for a subsequent appointment with the counsellor.

**Figure 4.**
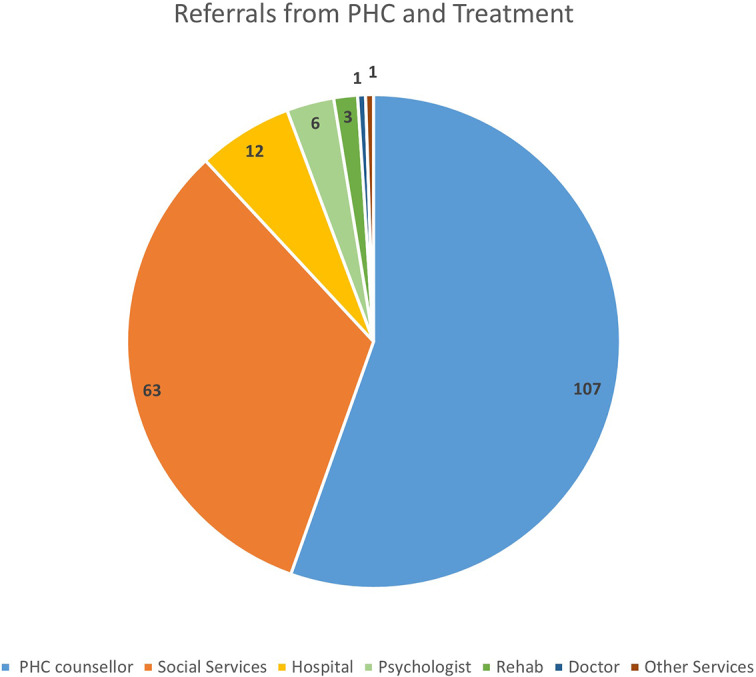
CMED referral types and treatment.

Eighty-six clients (39%) were referred by the Registered Nurse to services outside of the PHC facility (Figure 4). These referrals included social services (*n* = 63), hospital services (*n* = 12), psychologists (*n* = 6), substance rehabilitation centres (*n* = 3), doctor (*n* = 1) and other referrals (*n* = 1). Social referrals included referrals to social workers, the Department of Home Affairs for identity document applications and the Department of Social Development to access the South African Social Security Agency for social grant applications. Fifteen clients received more than one referral.

### Adoption

The variation in adoption at a facility level was calculated by dividing the number of CMED visits conducted by each WBPHCOT by the number of CHWs in each WBPHCOT (Table 3).

**Table 3.** CMED adoption per WBPHCOT Table 3. long description.

SUB-DISTRICT	WBPHCOT community catchment	# CHWs trained in CMED	# CMED visits	Average CMED administration per CHW per WBPHCOT
Dannhauser	One	24	785	33
Newcastle	Two	36	1,199	33
	Three	9	151	17

The highest adoption of the CMED occurred among CHWs from Facilities One and Two, which had an equal number of CMED administrations per CHW (33).

### Factors influencing high and low adoption

A common theme across the two high-adopting WBPHCOTs was the presence of engaged leadership. In each linked clinic, facility Operational Managers (OM) supported both general WBPHCOT activities and, more specifically, the implementation of the CMED intervention and referral pathways. WBPHCOTs One and Two also had supportive OTLs who attended mentorship activities alongside their CHWs. The OTL from WBPHCOT Two was well respected in the community, particularly by individuals with substance use challenges, while the OTL from WBPHCOT One ensured that clients referred were consistently attended to and followed up appropriately.

In the facility to which WBPHCOT One was linked, there were three trained lay counsellors. Two were trained from the outset, while a third, an enrolled nurse, was trained later in the intervention. The newly trained lay counsellor was also instrumental in introducing a client booking system to improve access to counselling services.

In contrast, WBPHCOT Two experienced challenges related to poor communication between the OTL, CHWs and the facility lay counsellor, which impacted coordinated implementation of the intervention and the availability of the lay counselling service at the facility. Similar challenges were observed in WBPHCOT Three, which had the lowest average number of referrals per CHW. In this facility, problems within the referral system between the WBPHCOT and the clinic were reported, and the lay counsellor was described as not consistently attending to referrals. Furthermore, although WBPHCOT Three initially had a supportive OTL who provided consistent mentorship to CHWs, the intervention faced challenges when the OTL was transferred to another facility midway through implementation. This disruption led to a loss of continuity, contributed to emerging tensions within the team, and disrupted adoption of the CMED intervention.

A common barrier across all three WBPHCOTs was the underdevelopment of data systems. CHWs manually recorded visits and progress reports in two-quire exercise books. Additionally, there was a lack of indicators and targets along the care cascade for any condition at a household level, making CQI initiatives challenging.

In relation to Maintenance, while we were unable to monitor continued use of the CMED by WBPHCOTs over time, the CMED had been adopted into policy and was being scaled up by the KZN Department of Health across the KZN province as well as adopted by the NDoH and the 2024 Community Health Worker Training Materials for Foundation Phase (https://knowledgehub.health.gov.za/elibrary/2024-community-health-worker-chw-training-materials-foundation-phase).

## Discussion

In the WBPHCOT sub-sample where the full cascade of Reach data was collected, CHWs administered the CMED to 15% of the total number of household visits made within a period of 6 months. As indicated by our previous research on the CMED, CHWs are often aware of mental health and social challenges present in their households, using their discretion as to which households they would administer the CMED depending on whether they thought it would be helpful to the household, with the tool providing them with a strategy to talk about possible mental health conditions in these families (Grant et al., 2023). Interestingly, the percentage of household members screened as being at-risk of having mental health conditions and referred to PHC facilities for further assessment and management (17%) reflects the national 12-month prevalence rate of common mental health conditions [16.5%] (Herman et al., 2009). Of those referred, over 60% presented at PHC facilities for further assessment, diagnosis and management. This finding is comparable to results from the Community Informant Detection Tool intervention in Nepal, from which the CMED intervention was adapted, where approximately two-thirds (67%) of individuals identified and referred through proactive community detection accessed health services (Jordans et al., 2017). These findings suggest that community-based case detection and referral approaches delivered by lay or community health workers may be effective in promoting help-seeking and linkage to mental health care in LMIC settings.

International literature suggests that understanding mental health conditions is important in driving help-seeking behaviour (van den Broek et al., 2023). Unlike the traditional symptom checklist approach to screening, which is divorced from promoting understanding of mental health conditions, the CMED tool used a vignette prototype matching approach, which contained culturally and contextually appropriate stories of characters with the symptoms of the various conditions (Grant et al., 2021). The feasibility study showed that this approach assisted household members to understand and self-identify symptoms of mental health conditions, assisting to promote help-seeking behaviour (Grant et al., 2023), notwithstanding contextual barriers to accessing care such as out-of-pocket expenditure on transport and long clinic waiting time (Grant et al., 2023).

As discussed in the introduction, WBPHCOTs are an essential component of re-engineering of PHC which foregrounds health promotion, prevention and empowering people and communities to be in control of their health. To this end, WBPHCOTs play an essential role. Notwithstanding the limitations of this study, the findings from the real-world data presented in this paper demonstrate the potential of the WBPHCOT-delivered CMED tool to narrow the mental health treatment gap in South Africa, through strengthening understanding of mental health symptoms, risk screening and help-seeking at the household level.

In keeping with the purpose of implementation science, the data also highlight a number of barriers and facilitators to the optimal delivery of the CMED tool. From a health systems perspective, both the ‘hardware’ – including policies, information systems and resources – and the ‘software’, such as leadership, supervision and organisational culture, shape the implementation of innovations (Burger and Gilson, 2025).

In terms of hardware, at the time of the study, WBPHCOT information systems were under-developed, with teams still manually recording information on household visits. The lack of household-level indicators and targets along the care cascade and the absence of digitalised reporting systems for community-based services reduced the priority afforded to the work of WBPHCOTs (Whidden et al., 2018). Health systems are often performance driven with managers prioritising activities against which their facilities are measured. Evidence suggests that the implementation of innovations within health systems is supported by robust, data-driven quality improvement systems (Bhardwaj et al., 2014). This performance orientation influences not only day-to-day decision making but also the allocation of resources and budgets, with funding and support typically directed towards services linked to established indicators and targets (Petersen, 2021). As a result, activities that are not routinely measured – such as aspects of WBPHCOT service delivery at the household level – may be deprioritised and under-resourced. Further, this limits data-driven CQI processes aimed at tracking and improving WBPHCOT services along the care cascade. In the case of the CMED intervention, limited household-level monitoring and reporting systems constrained opportunities for data-driven adaptation, supervision and CQI across the care cascade within all nine community catchment areas.

More broadly, the organisation and monitoring of health systems through verticalised programmes and condition-specific indicators may act against integrated models of care at the point of service delivery, including the integration of mental health within PHC. Where management, supervision, and reporting systems are structured around siloed programmes and targets, healthcare personnel may prioritise activities linked to measurable indicators over integrated, person-centred care. In this context, leadership operates not only as an individual competency but also as a mechanism through which competing system priorities are negotiated and translated into practice at facility and community levels.

Supportive leadership and supervision emerged as strong facilitators of adoption by the high-adopting WBPHCOT teams, at both OM and OTL levels. These findings align with the ‘software’ elements of the health system, highlighting the importance of supportive relationships and organisational climate enabling implementation. Evidence consistently shows that the quality of CHW supervision influences programme effectiveness, CHW motivation, and CHWs’ relationships with and credibility among PHC facility-based staff (Assegaai and Schneider, 2019). Our findings suggest that engaged leadership facilitated implementation of the CMED intervention by strengthening referral pathways, supporting innovations in service delivery and enhancing the credibility of WBPHCOTs within the community and facility. Conversely, disruptions in leadership negatively affected implementation. While leadership skills can be developed (Phillipson et al., 2025) and should be included in induction and in-service training programmes for healthcare personnel taking up such supervisory roles, it should be recognised that, given resource constraints, many OTLs have competing demands and must balance clinical and supervisory responsibilities.

Additionally, facility-level leadership operates within, and is shaped by, the broader policy and governance environment. The South African NDoH developed the WBPHCOT strategy, which outlines roles and responsibilities, implementation guidelines and the CHW curriculum (Schneider et al., 2018). Detailed design, implementation and funding arrangements are subsequently determined at the provincial level, contributing to variation in how WBPHCOT services are implemented and supported across the health system (Schneider et al., 2018). In their review of official WBPHCOT policy documents and guidelines, Assegaai and Schneider (2019) found that although supervision and support were emphasised, the absence of a coherent supervision framework made the operationalisation of WBPHCOT supervision challenging. These policy structures (hardware elements) determine how services are organised and monitored, while their implementation is mediated by leadership at provincial, district and facility levels. Our findings add to the growing calls for improved governance frameworks for WBPHCOTs that ensure support, recognition and accountability, and that more effectively translate policy into consistent implementation, thereby reinforcing the recognition and value of WBPHCOTs within the health system (Assegaai and Schneider, 2019; Schneider, 2019; Buthelezi et al., 2025).

Another important barrier to adoption of the CMED tool by WBPHCOTs, which was also identified in the initial feasibility study (Grant et al., 2023), was the lack of consistent mental health services, particularly counselling services, to meet the demand generated. Strengthening demand for services needs to be balanced with a sufficient supply of services which is an important concern to be considered for widespread scale-up of the CMED tool. Over half of the referred household members who presented at PHC facilities were referred onwards to the co-located counselling service. This underscores the importance of such a co-located counselling service at PHC facilities, with a strong evidence base for task sharing of evidence-based counselling approaches globally and nationally (Singla et al., 2017; Myers et al., 2022). Such a service is particularly important within the South African context, where professional PHC nurses are not authorised to initiate psychotropic medication, requiring PHC doctors who provide sessional services to PHC facilities to initiate such medication, with indications of only one such referral. Almost half of the referrals beyond the PHC facilities included social referrals. These findings are understandable given the well-known social determinants of mental health conditions. Only 12% of onward referrals outside of the PHC facilities were to the district hospital or other specialist services. These findings suggest that the widespread scale-up of the CMED is unlikely to burden specialist services but will assist to close the treatment gap at the PHC level. This is in line with the World Health Organisation optimal model of mental healthcare, where the bulk of services are provided at the community and PHC levels (World Health Organization, 2009), and informs the national South African Mental Health Policy Framework and Action Plan 2023–2030 (National South African Department of Health, 2023).

### Limitations

The analysis was limited by our reliance on real-world data, which varied in completeness and consistency across clinics, with logistical and financial constraints preventing the collection of parallel research data to verify routine CHW self-reported data. Complete care cascade data were available for only three of the nine original community catchment areas and their associated WBPHCOTs. This may have introduced selection bias, as facilities with more complete routine data systems and stronger implementation processes may also have been more likely to demonstrate higher levels of intervention uptake. Despite this limitation, 14,262 household visits were conducted across the three community catchment areas, of which 2,135 received the CMED intervention during the 6-month study period, indicating that the findings were drawn from implementation at substantial scale. In addition, the variation observed across the three WBPHCOTs in leadership, referral coordination and intervention adoption suggests that the included sites reflected differing implementation contexts rather than only high-performing settings. Nevertheless, the findings should be interpreted cautiously, and further research across a broader range of implementation contexts is required to better understand the generalisability of these findings across WBPHCOTs and PHC settings in South Africa.

## Conclusion

The real-world data used in this study indicate that the task-sharing WBPHCOT-delivered CMED tool within households has potential to narrow the treatment gap in South Africa and potentially other LMICs through early identification and linkage to care at the community level. The WBPHCOT system is being rolled out in South Africa and provides a viable CHW platform for the delivery of mental health services focused on demand generation within communities using the CMED. CHWs also play an important role in the healthcare systems of many LMICs. As noted, such demand generation needs, however, to be balanced with an adequate supply of services. In the absence of this, trust in the health system by service users, including WBPHCOTs, can be compromised. Further, the CMED tool is layered into the existing services provided by WBPHCOTs. For its potential to be realised at scale, the need for stronger governance and accountability structures of the WBPHCOT system has been highlighted. In particular, it is the need for strengthening routine data systems, including improved monitoring and evaluation processes, strengthened supervisory capacity, routine household-level indicators and investment in integrated electronic reporting systems.

## Supporting information

10.1017/gmh.2026.10256.sm001 Grant et al. supplementary material Grant et al. supplementary material

## Data Availability

The data that support the findings of this study are available from the corresponding author, MG, upon reasonable request.

## Supplementary material

The supplementary material for this article can be found at http://doi.org/10.1017/gmh.2026.10256.

## Long descriptions

### Figure 1. Long description

The comic consists of four panels numbered 5 through 8. In each panel, two women sit at a table with an open book. Between them is a vertical thermometer with a green bulb at the base and a gradient rising through yellow and orange to red at the top.

Panel 5. Anna, on the left, says, Yes, you see the green is when we can cope with the normal stress of life. Mpumi, on the right, asks, And does that mean we can still do our jobs properly even if we are stressed?

Panel 6. Anna replies, Yes, that is right and it also means that our behaviour is still ok and it does not affect us and those around us negatively! Mpumi responds, I see, that is what you said earlier... things happen in our lives that can affect the way we think, feel and act. A green ring circles the lower green section of the thermometer.

Panel 7. Anna says, Sometimes when too much happens in our lives, we can struggle to think clearly and it can affect the way we feel and then that affects how we behave. Mpumi asks, So the orange is when everything starts to become too much and we begin to struggle to cope? An orange ring circles the middle section of the thermometer.

Panel 8. Anna explains, Yes, Mpumi, and when we are in the red zone we cannot cope and it negatively affects how we do our normal daily activities. For example things like see/speak to friends and family, cook, wash or dress properly, clean the house, shop, manage money and work. Mpumi concludes, Yes, Anna this is helping me to understand that our mental health changes during our lifetime. A red ring circles the top red tip of the thermometer.

Navigate back to Figure 1..

### Figure 2. Long description

A vertical informational poster divided into sections.

Header: A dark green banner asks if the reader has little interest in doing things or feels hopeless, stating that if yes, they might have DEPRESSION. A small box in the corner says COMMUNITY.

Symptoms Section: Titled People who are depressed often, followed by three colored horizontal bands with icons.

* Yellow band with a THINK icon: Lists finding it hard to concentrate, negative thoughts about the future, and thoughts of self-harm.

* Purple band with a FEEL heart icon: Lists feeling sad, worthless, hopeless, and tearful.

* Teal band with an ACT icon: Lists losing interest, problems with sleeping or eating, and avoiding social contact.

Help Section: Titled Help yourself by, followed by four numbered green boxes.

1. Doing these things, like: Something you enjoy (illustrated by gardening), Exercising (illustrated by jump rope), and Talking to a friend (illustrated by two people sitting under a tree).

2. Watching this video: Includes a Q R code and a YouTube link.

3. Calling SADAG a free counselling hotline: Lists three phone numbers: 0800 2122 23, 0800 7080 90, and 0800 4567 89.

4. Telling the C H W and getting a referral to the clinic for counselling and/or treatment.

Footer: A dark green banner on the left says Remember, you can come through this – most people do! The right side contains logos for the Health Department Province of KwaZulu-Natal and credits to MhINT, ASSET, University of KwaZulu-Natal, and I-TECH 2021.

Navigate back to Figure 2..

### Table 1. Long description

The table consists of three columns: W B P H C O T, Number of O T Ls, and Number of C H Ws.

* W B P H C O T One asterisk: 1 O T L, 24 C H Ws.

* W B P H C O T Two asterisk: 2 O T Ls, 36 C H Ws.

* W B P H C O T Three asterisk: 1 O T L, 9 C H Ws.

* W B P H C O T Four: 4 O T Ls, 9 C H Ws.

* W B P H C O T Five: 7 O T Ls, 100 C H Ws.

* W B P H C O T Six: 4 O T Ls, 6 C H Ws.

* W B P H C O T Seven: 3 O T Ls, 15 C H Ws.

* W B P H C O T Eight: 3 O T Ls, 18 C H Ws.

* W B P H C O T Nine: 2 O T Ls, 28 C H Ws.

* Totals: 27 O T Ls and 245 C H Ws.

The asterisk denotes facilities included to evaluate reach.

Navigate back to Table 1..

### Table 2. Long description

The table is divided into two main sections: Referrals from community to P H C facility level and Onward referrals from the P H C nurse.

Section 1: Referrals from community to P H C facility level

* Total household visits: Started at 1,911 in Feb 22, peaked at 2,702 in May 22, with a total of 14,262.

* C M E D visits: Ranged from 238 to 468 per month, totaling 2,135 (15 percent of visits).

* Clients referred using C M E D: Increased significantly in July 22 to 159 (49 percent), with a total of 360 (17 percent).

* Clients presenting at clinics after referral: Totaled 222 (62 percent of referrals).

Section 2: Onward referrals from the P H C nurse

* Clients referred to the P H C counsellor: Totaled 107 (48 percent).

* Clients referred to social services: Totaled 63 (28 percent), with a peak of 33 in July 22.

* Clients referred to the hospital: Totaled 12 (5 percent).

* Psychologist: Totaled 6 (3 percent).

* Rehab: Totaled 3 (1 percent).

* Doctor: Totaled 1 (0.45 percent).

* Other: Totaled 1 (0.45 percent).

Navigate back to Table 2..

### Figure 3. Long description

The diagram consists of four horizontal rounded bars of decreasing width stacked vertically, representing a care cascade. A legend on the left side maps colors to specific stages.

* Top layer (Dark Blue): Number of total C H W visits. The bar contains the number 14262.

* Second layer (Orange): Number of C M E D visits conducted. The bar contains 2135 (15 percent).

* Third layer (Green): Clients referred to the P H C facility using C M E D. The bar contains 360 (17 percent).

* Bottom layer (Grey-Blue): Clients presenting at the P H C facility after referral. The bar contains 222 (62 percent).

Navigate back to Figure 3..

### Table 3. Long description

The table consists of five columns: SUB-DISTRICT, W B P H C O T community catchment, number of C H W s trained in C M E D, number of C M E D visits, and Average C M E D administration per C H W per W B P H C O T.

* Row 1: Dannhauser sub-district, Catchment One. 24 C H W s trained, 785 visits, average of 33 administrations.

* Row 2: Newcastle sub-district, Catchment Two. 36 C H W s trained, 1,199 visits, average of 33 administrations.

* Row 3: Newcastle sub-district, Catchment Three. 9 C H W s trained, 151 visits, average of 17 administrations.

Navigate back to Table 3..
