# Supplementary material for: Helping to improve demand for mental health services: Reach and adoption of a community mental health education and detection tool by community health workers within routine care in South Africa
Source: Glob Ment Health (Camb). 2026 Jun 24;13:e140. doi: 10.1017/gmh.2026.10256 (PMC13373268; doi:10.1017/gmh.2026.10256)
Supplement: Grant et al. supplementary material [file S2054425126102568sup001.docx]

**Supplementary File:** **Community Mental Health Education and Detection (CMED) Tool**

| **1. Brief Name** | Community Mental Health Education and Detection (CMED) Tool | | | | | |
| --- | --- | --- | --- | --- | --- | --- |
| **2. Why** | The CMED tool was developed as part of a broader implementation research study, the Southern African Research Consortium for Mental health INTegration (SMhINT), which used implementation science to evaluate the scale up of the Mental health INTegration (MhINT) collaborative care package.  The South African National Department of Health (NDoH) and the KwaZulu-Natal Department of Health (KZN DoH) requested the MhINT team to develop and validate a mental health tool that could be used at the community level by ward-based primary health care outreach teams (WBPHCOTs) to identify community members with possible mental health conditions (depression, anxiety, psychosis, harmful alcohol and drug use) during their routine household visits and facilitate linkage to care. Given the low levels of mental health literacy in South African communities as well as the screening and health promotion role of WBPHCOTs it was agreed that mental health promotion (increasing mental health awareness, psychoeducation, and self-care) be included as part of the CMED tool.  The continuous quality improvement processes embedded within MhINT also identified the need to strengthen mental health awareness, mental health risk screening and linkage to care at a community level.  WBPHCOTs are ideally comprised of a professional nurse/enrolled nurse as supervisor known as an Outreach Team Leader (OTL) and a team of approximately 8-10 CHWs. WBPHCOTs provide household-level community care and help bridge the gap between communities and primary health care facilities. | | | | | |
| **3. What (materials)** | The CMED tool and related training materials were developed to support the training of CHWs in mental health and the delivery of the CMED tool. The training resources for each level of the cascade form a coherent suite of materials designed to capacitate end users to provide comprehensive and standardised mental healthcare within the health system. | | | | | |
|  |  | Community screening tool | | Community Mental Health Education and Detection (CMED) Tool: available in English and *isiZulu*. The CMED is designed for use by WBPHCOTs and is based on a prototype matching approach, in which symptoms of mental health conditions are presented in five case vignettes (prototype paragraphs) with related illustrations. These are used to facilitate the detection of possible depression, anxiety, psychosis, harmful alcohol and drug use, by matching the presenting symptoms of household members to the relevant prototype paragraph. Psychoeducation is also embedded within each case vignette.  The concept of mental health is first introduced through a vignette using the analogy of an Emotional Health Thermometer to help household members understand that mental health exists on a continuum, ranging from optimal wellbeing to low levels of wellbeing and difficulty functioning.  The CMED includes a Mental Health Question Flowchart containing a set of guiding questions to help CHWs determine which vignette to administer within the household.  CHWs then read the selected case vignette. Guiding questions and discussion prompts help CHWs to ask questions that initiate discussion and guide the family members to self-identify where applicable. A set of structured questions also guides the CHWs in making decisions regarding onward referral to the PHC facilities.  Each vignette is followed by healthy lifestyle information on what community members can do at home for their mental health. | | |
|  |  | Training materials | | The training was structured around the following resources:   - **Part One:** Foundation Phase Community Health care Worker Self-Care and mental health training in the CMED tool - **Part 2:** Work Integrated Learning (WIL)   Separate training materials were developed for:   - Master Trainers (Training of the trainer (ToT) manual) - Outreach Team Leaders (Facilitator manual) - Community Health Workers (User manual) | | |
| **4. What - procedures** | OTLs from nine WBPHCOTs servicing nine catchment areas in the Amajuba District were trained and mentored by the intervention team (UKZN Master Trainers) to deliver CMED training to CHWs. CHWs were subsequently trained by their OTLs with support from the intervention team. Following the training, CHWs were accompanied on household visits by their OTL and a UKZN Master Trainer. During these visits, CHWs had the opportunity to administer the CMED tool to households and received additional mentorship on tool delivery and referral processes.  OTLs also received training in Work Integrated Learning (WIL), delivered by the intervention team and DoH trainers, in which concepts first introduced during the foundation training were reinforced and expanded upon. Additional sessions included grief and loss, mental health concerns among older adults and responding to self-harm in the community. The programme also incorporated self-care and leadership skills for OTLs, ongoing mentorship and support, and data monitoring for quality improvement to strengthen OTL support for CHWs.  Referral pathways were identified during the continuous quality improvement process as a bottleneck limiting patient access to mental health care. In response, the implementation team and the DoH District mental health coordinator worked with facility operational managers, counsellors, OTLs, and CHWs to strengthen referral documentation and improve access to care following CMED referrals.  Extensive engagement with the NDoH and the KZN DoH, together with the co-production of the CMED tool and training materials, contributed to a “goodness of fit” of the community tools within the existing health system. | | | | | |
| **5. Who** | a. Who Provided   - The CMED is designed to be delivered at a household level by CHWs. - The WIL training is designed to support OTLs in mentoring CHWs in their delivery of the CMED tool and facilitation of mental health referrals at a community level. - Leaflets are also distributed by CHWs. | | | | | |
|  | b. Who Received   - Community members receive the CMED tool and accompanying leaflets. | | | | | |
| **6. How and where** | The CMED and leaflets are delivered face-to-face to household members in their homes, as well as in community venues such as well child centres. | | | | | |
| **7. When and how much** | - The CMED tool is delivered to households based on needs identified by CHWs using the flowchart, as well as for general health promotion purposes. - Leaflets are distributed by CHWs at their discretion during household visits when an appropriate condition is identified through the use of the flowchart and CMED vignettes. | | | | | |
| **8. Tailoring** | The CMED has undergone extensive tailoring through engagement with the NDoH and KZN DoH management, as well as through the research process, including the feasibility study involving both WBPHCOTs and household members. As a result, the CMED has evolved to version 33.  New content was introduced during the scale-up of the CMED to address gaps identified in the original material. This included content on supporting older adults with memory loss (dementia) and guidance on adherence treatment for common NCDs such as HIV, TB and diabetes. A stepped-up approach to health promotion was also incorporated. In addition to the healthy lifestyle information, households received links to self-help and problem management skills, counselling hotlines and guidance on where to access support within the healthcare system. | | | | | |
| **9. How well-planned? /**  **How well actual** | **Target** | | **Activity details** | | **Dosage planned** | **Dosage actual** |
|  | Master trainers | | ToT foundational training | | Half day training planned | Half day training completed |
|  |  |  | ToT WIL training | | Half day training planned | Half day training completed |
|  | OTLs | | Facilitator foundational training | | One day training planned | One day training completed |
|  |  |  | WIL facilitator training | | 1 x One day training workshops planned | 1 x One day training sessions completed |
|  | CHWs | | User foundational training | | 9 x Two day training workshops planned | 9 x Two day training sessions completed |
|  | WBPHCOT team (OTLs and CHWs) | | Mentorship of CHWs following Part One foundational training | | 9 x mentorship visits | 13 x mentorship visits completed |
|  |  |  | Support visits following training | | 40 x support visits planned | 49 x support visits completed |
| **10. Modification** | Through consultation with the NDoH, the WIL training was added to align with the existing training approach used for CHWs in other disease areas. This alignment was necessary to support adoption of the CMED and training at a national level as part of community-level care.  Content relating to older adults with memory loss and confusion was added to support families caring for a family member with dementia, in response to requests from CHWs and OTLs.  The depression, anxiety and loss and grief leaflets were developed to support delivery of the CMED within households, particularly in the context of the COVID-19 pandemic. | | | | | |
