# Supplementary material for: Helping to improve demand for mental health services: Reach and adoption of a community mental health education and detection tool by community health workers within routine care in South Africa
Source: Glob Ment Health (Camb). 2026 Jun 24;13:e140. doi: 10.1017/gmh.2026.10256 (PMC13373268; doi:10.1017/gmh.2026.10256)
Supplement: Peer review history

# Reviewer report: Review: Helping to improve demand for mental health services: Reach and adoption of a community mental health education and detection tool by community health workers within routine care in South Africa — R0/PR2

**DOI:** 10.1017/gmh.2026.10256.pr2

This manuscript presents a timely and policy relevant implementation evaluation of the Community Mental Health Education and Detection (CMED) tool delivered by community health workers (CHWs) in South Africa. The study addresses a critical and underexplored challenge in global mental health: strengthening demand side mechanisms for mental health care within low and middle income country (LMIC) health systems. The authors adopt an implementation science lens and draw on real world data, making the findings highly relevant for scale up and health systems strengthening. Overall, the study makes a strong contribution to the literature; however, several conceptual, methodological, and interpretive issues warrant attention to strengthen clarity, rigor, and generalizability.

Although the RE AIM framework is referenced, the analysis primarily focuses on Reach and Adoption, with minimal engagement with Effectiveness, Implementation fidelity, or Maintenance. While the authors acknowledge this scope, the manuscript would benefit from a clearer conceptual justification for limiting analysis to these two domains and a more explicit discussion of how findings inform longer term sustainability and maintenance.

A significant methodological limitation is that complete care cascade data were available for only three out of nine WBPHCOTs. While the authors are transparent about this constraint, the implications for interpretation merit deeper discussion. It remains unclear to what extent the three facilities with complete data are representative of the broader program context, and selection bias may have influenced estimates of reach, referral completion, and adoption.

The use of CHW reported routine data introduces potential reporting bias, inconsistencies, and missingness, which could affect estimates of screening rates and referral follow through. Although these limitations are acknowledged, the manuscript would benefit from a more critical discussion of how data quality challenges may have influenced key findings and from clearer strategies for strengthening data systems in future scale up efforts.

The manuscript demonstrates successful detection and referral; however, it provides limited insight into clinical outcomes or patient level benefits beyond initial assessment and onward referral. While acknowledging that effectiveness was not a primary outcome, readers would benefit from clearer articulation of how the CMED tool fits within a broader theory of change linking detection, care engagement, and improved mental health outcomes.

While leadership and supervision emerge as key facilitators, the analysis of adoption variability remains largely descriptive. A deeper engagement with implementation theory—such as mechanisms of leadership influence, organizational readiness, or contextual constraints—could strengthen the explanatory power of the qualitative findings and enhance transferability to other settings.

The extensive supplementary materials, while informative, are highly detailed and at times repetitive. Greater synthesis or selective streamlining—particularly of training schedules and manuals—could improve readability and help readers focus on the intervention components most central to implementation success.

This manuscript presents a substantively important and well executed implementation evaluation that meaningfully advances understanding of demand side mental health interventions delivered by CHWs in LMIC contexts. Its strengths lie in its policy relevance, contextual grounding, and use of real world implementation data. Addressing the methodological limitations related to data completeness, deepening engagement with implementation theory, and strengthening the discussion of implications for sustainability would further enhance its contribution.

# Reviewer report: Review: Helping to improve demand for mental health services: Reach and adoption of a community mental health education and detection tool by community health workers within routine care in South Africa — R0/PR3

**DOI:** 10.1017/gmh.2026.10256.pr3

**Authors:** Simone Honikman

**Affiliations:** Psychiatry and Mental Health, University of Cape Town, South Africa

This is a strong and highly relevant contribution to the global mental health literature. Using an implementation science framework, the authors assess the reach and adoption of a previously validated mental health tool (CMED) delivered by community health workers within a low-resource South African setting. The paper is well written, the methods are detailed, robust, and appropriate, and the discussion is thoughtful and engaging. The study generates several novel and useful insights for South Africa as well as for other comparable contexts.

I have a few minor suggestions that may strengthen the manuscript further.

Introduction

• The reasons presented for why individuals experiencing mental health symptoms may not seek care are somewhat limited. At present, the framing appears to emphasise individual-level barriers, without sufficient attention to the ways in which the symptoms of mental health conditions themselves, as well as structural factors, may constrain help-seeking. In particular, limited access to appropriate and responsive services should also be acknowledged. Although this is raised in the discussion, it would be helpful to signal it earlier in the introduction as well.

Methods

• In the description of CHW training, the reference to the choice of mental health session is somewhat unclear. This may relate to a broader mental healthcare process described in the cited source, but additional context within this paper would help the reader.

• I suggest revising the long sentence on page 8, lines 33–40, for clarity.

• Regarding mentoring and support, was a fidelity checklist used? If not, it may be worth acknowledging this as a possible limitation.

• Please clarify whether the WIL applied to outreach team leaders, community health workers, or both.

Discussion

• It would be valuable to contextualise the finding that 60% of referred participants presented at PHC facilities by comparing this level of uptake with referral completion or service uptake reported in other primary mental healthcare settings.

• It may also be useful to consider whether contextual factors unrelated to the CMED intervention itself, such as geography, transport access, or perceptions of facility quality and reputation, may have influenced this finding.

• In the limitations section, the final sentence could be revised for grammatical clarity.

# Editor report: Recommendation: Helping to improve demand for mental health services: Reach and adoption of a community mental health education and detection tool by community health workers within routine care in South Africa — R0/PR4

**DOI:** 10.1017/gmh.2026.10256.pr4

**Affiliations:** Narodni ustav dusevniho zdravi, Czech Republic

Thank you for submitting your manuscript titled: “Helping to improve demand for mental health services: Reach and adoption of a Community Mental health Education and Detection tool by community health workers within routine care in South Africa.” to Cambridge Prisms: Global Mental Health. Both reviewers agree this is a study with high policy relevance and a great contribution to the implementation science literature in LMIC contexts. Reviewers have also pointed out possible improvements to the manuscript, especially in terms of the discussion of the methodology and deeper engagement with the data. To move forward, please submit a revised version of your manuscript and a point-by-point response addressing the reviewers' comments.

# Editor report: Decision: Helping to improve demand for mental health services: Reach and adoption of a community mental health education and detection tool by community health workers within routine care in South Africa — R0/PR5

**DOI:** 10.1017/gmh.2026.10256.pr5

**Affiliations:** Johns Hopkins University Bloomberg School of Public Health, United States

No accompanying comment.

# Reviewer report: Review: Helping to improve demand for mental health services: Reach and adoption of a community mental health education and detection tool by community health workers within routine care in South Africa — R1/PR7

**DOI:** 10.1017/gmh.2026.10256.pr7

**Authors:** Simone Honikman

**Affiliations:** Psychiatry and Mental Health, University of Cape Town, South Africa

Thank you, I am satisfied with the revisions made and have no further comments.

# Editor report: Recommendation: Helping to improve demand for mental health services: Reach and adoption of a community mental health education and detection tool by community health workers within routine care in South Africa — R1/PR8

**DOI:** 10.1017/gmh.2026.10256.pr8

**Affiliations:** Narodni ustav dusevniho zdravi, Czech Republic

The revisions successfully address all points raised during the review process, and the manuscript is now suitable for publication in its current form.

# Editor report: Decision: Helping to improve demand for mental health services: Reach and adoption of a community mental health education and detection tool by community health workers within routine care in South Africa — R1/PR9

**DOI:** 10.1017/gmh.2026.10256.pr9

**Affiliations:** Johns Hopkins University Bloomberg School of Public Health, United States

No accompanying comment.
